# Training needs in metabolomics

**DOI:** 10.1007/s11306-015-0815-6

**Published:** 2015-05-29

**Authors:** Ralf J. M. Weber, Catherine L. Winder, Lee D. Larcombe, Warwick B. Dunn, Mark R. Viant

**Affiliations:** School of Biosciences, University of Birmingham, Edgbaston, Birmingham, B15 2TT UK; Department of Physiology, Anatomy & Genetics, University of Oxford, Oxford, OX1 3PT UK

## Introduction

Metabolomics has come of age and is predicted to have a compound annual growth rate of 30 % from 2014 to 2019 and a market value of $2.1 billion by 2019 (Rohan 2014). While recent progress has been driven by technological advances in both analytical instrumentation and software, and by the availability of ring-fenced research funding in some countries, insufficient attention has focused on developing the specialist training required to support this growth. The international Metabolomics Society was established to promote the growth of this field (Goodacre 2005), to stimulate collaboration among scientists in academia, government and industry, and to deliver conferences and training workshops. ELIXIR-UK (http://elixir-uk.org/) is the United Kingdom node within the European ELIXIR infrastructure and currently focuses on bioinformatics training provision that will be delivered in specialised centres, through face-to-face courses and e-learning; this training will be in partnership with other European ELIXIR Nodes. To guide the development (and ultimately provision) of fit-for-purpose training, considering factors such as scientific content, expertise levels of attendees, knowledge level of course, and mechanisms by which to provide the training, the international Metabolomics Society and ELIXIR-UK have jointly conducted a global assessment of the training needs in metabolomics science. Specifically, a questionnaire was developed and widely distributed to determine the training needs of our international community, the training programs that already exist, the new course content to develop and the mechanisms by which this training could be delivered. By “metabolomics science”, we include the underpinning science and technology (experimental design, analytical measurements, computational analyses and informatics) and any field of application from microbes to plants and animals, including humans. Here we report the results of the training needs questionnaire, from which we have derived a series of key recommendations to support the training of the growing metabolomics community. By sharing the full results, we hope that others will conduct additional analyses and be able to derive further interesting conclusions, and collectively help to guide the development of metabolomics training programs globally.

## Results questionnaire

The training needs questionnaire was made available online, using Survey Monkey (https://www.surveymonkey.com/, see supplementary information for full survey results (SI1) and http://metabolomicssociety.org/training-needs-in-metabolomics), for a period of ca. 7 weeks (1st September to 20th October 2014). The questionnaire, comprising of 37 questions, was promoted through multiple channels including MetaboNews (http://www.metabonews.ca), websites (e.g. Metabolomics Society—http://metabolomicssociety.org/), multiple mailing lists, and metabolomics-related meetings and workshops. A total of 202 responses from 36 countries spanning six continents were received (Background dashboard Figure SI2-3 or http://metabolomicssociety.org/training-needs-in-metabolomics). Here we summarise the most important findings, while the complete set of responses (excluding personal identifiers) are available in full as electronic supplementary material.

The majority of respondents comprised of academics (>75 %), at different career levels, from a wide range of scientific remits (including plant science, clinical science, environmental science and nutritional science) and sample types (Background dashboard Figures SI2-1, SI2-4, SI2-6 & SI2-7). More than 50 % of the respondents considered themselves to have up to 4 years of experience in the field of metabolomics (0–2 years 29 %; 2–4 years 23 %), whereas approximately 45 % have over 4 years of experience (Background dashboard Figure SI2-5). 65 % of the responder’s daily work consists of a combination of wet laboratory work and data handling/processing (Background dashboard Figure SI2-2). Taken together, the responses to these questions clearly indicate that the respondents have a wide range of interests, a wide range of experience, and span both the analytical and computational sciences, suggesting the conclusions that we draw below are widely applicable to the community.

Respondents requested that training should span a wide range of analytical and bioinformatics disciplines and cover both introductory and advanced training requirements. The most frequently requested training in analytical laboratory related topics were (a) spectral interpretation and metabolite identification, and (b) data quality (including experimental design, QC samples and QA procedures) (Training needs dashboard Figure SI3-1 or http://metabolomicssociety.org/training-needs-in-metabolomics). This is perhaps not surprising as metabolite identification and data quality are currently “hot topics” within metabolomics. For laboratory-based subjects there is generally a preference for advanced training courses with the exceptions being for introductory courses in metabolic flux analysis, capillary electrophoresis and NMR spectroscopy. A large percentage of the respondents apply untargeted (77 %) and targeted (63 %) approaches in their analytical experiments (Background dashboard Figure SI2-8). While introductory training courses are required in targeted and untargeted approaches (to train new researchers in the field), the greatest demand is for advanced training courses within these areas (untargeted 79 % and targeted 78 %—Training needs dashboard Figure SI3-1).

There is a strong preference for advanced training *versus* introductory courses in bioinformatics related topics. However, the opposite is true for programming, where for almost all subjects basic training is preferred (Training needs dashboard Figure SI3-2). By far the majority of respondents use Microsoft Excel (78 %) in their research, but have limited confidence when applying more powerful bioinformatics tools (Background dashboard Figure SI2-10). The top priority for training provision in programming includes introductory courses in R, data mining and Matlab (Training needs dashboard Figure SI3-3). Within the bioinformatics topics the majority of respondents routinely apply data processing (81 %), biostatistics and chemometrics (78 %) and metabolite identification (74 %) in their research (Background dashboard Figure SI2-9). Arguably one of the most striking observations concerns the use of data repositories (such as MetaboLights in the EU and Metabolomics Workbench in the US). It is notable that the number of metabolomics datasets in these public repositories is growing at a very encouraging rate (http://metabolomexchange.org) given they are relatively new resources, yet only ca. 29 % of respondents have used databases (22 %) or data repositories (7 %) in their research (Background dashboard Figure SI2-9 and Training needs dashboard Figure SI3-4). Hence we can conclude that there is a clear need for further training courses to support the use of these important repositories, as recognised and requested by the respondents (Training needs dashboard Figure SI3-5). Furthermore, it suggests that these resources will have to be expanded to support the inevitable increase in their use, in particular as scientists seek to meet the data sharing policies of funding agencies and many publishers.

The preferred mechanism of training delivery is via face-to-face courses and ideally over a duration of 3 days (61 % of respondents). To develop and deliver this type and level of training, across multiple courses per year to meet demand, would require dedicated trainers. Currently few funding mechanisms exist to support the growth of a national or international community of trainers. A third of all respondents preferring to attend formally accredited training courses. Additionally, 59 % would more likely attend a course when it is organised by a well-recognised organisation, such as the Metabolomics Society, ELIXIR-UK and/or the Royal Society of Chemistry. Furthermore, a considerable fraction of respondents (46 %) would like more vendor-led training programmes. Industry-academia partnerships are currently driving innovations within the metabolomics field through the sharing of ideas and expertise. The extension of these partnerships to address the online and face-to-face training requirements within the metabolomics community could provide courses demonstrating the newest tools and technologies, and ensure the courses are updated to cover “hot topics”.

Approximately 43 % of respondents could name at least one existing face-to-face metabolomics course (courses listed in supplementary data—full survey results, see Supplementary Information and http://metabolomicssociety.org/training-needs-in-metabolomics), of which only half of these respondents had attended the course they named. Clearly there is a need to improve the advertising and potentially the accessibility of current and newly developed training courses, for example via Global Organisation for Bioinformatics Learning, Education & Training (GOBLET, http://mygoblet.org/) (Corpas et al. 2015), Training eSupport System (TeSS, https://tess.oerc.ox.ac.uk) and/or the Metabolomics Society (http://metabolomicssociety.org/). Online training is currently under utilised within metabolomics and could provide training courses that are globally accessible.

## Key recommendations

Our recommendations, of which the first three are the most important, comprise:

- Urgently develop a series of face-to-face and e-learning training courses to fill the knowledge gaps in analytical metabolomics and bioinformatics, as identified by the training questionnaire;
- Create new funding opportunities to build national and international networks of trainers to both develop and deliver training programs, thereby maximising existing investments by the funding agencies into research projects and facilities;
- Improve the advertising and accessibility of both existing and new training courses, for example via the ELIXIR TeSS portal (https://tess.oerc.ox.ac.uk) and Metabolomics Society network;
- To initiate discussions within the international metabolomics community to consider a formal accreditation of training programs in order to achieve high training standards, harmonisation across courses and the on-going redevelopment of courses as technologies evolve.

## Electronic supplementary material

Supplementary material 1 (XLS 135 kb)

Supplementary material 2 (PDF 146 kb)

Supplementary material 3 (PDF 200 kb)

## References

[CR1] Corpas M (2015). The GOBLET training portal: a global repository of bioinformatics training materials, courses and trainers. Bioinformatics.

[CR2] Goodacre R (2005). Metabolomics—the way forward. Metabolomics.

[CR3] Rohan (2014). Metabolomics Market worth $2,100 Million by 2019 (http://www.marketsandmarkets.com/PressReleases/metabolomics-technology.asp). In: Markets and Markets. http://www.marketsandmarkets.com/PressReleases/metabolomics-technology.asp.

