# Supplementary material for: Training needs in metabolomics
Source: Metabolomics. 2015 May 29;11(4):784–6. doi: 10.1007/s11306-015-0815-6 (PMC4475540; doi:10.1007/s11306-015-0815-6)

Metabolomics Survey - Background Questions (n=202)

1. Area of Employment

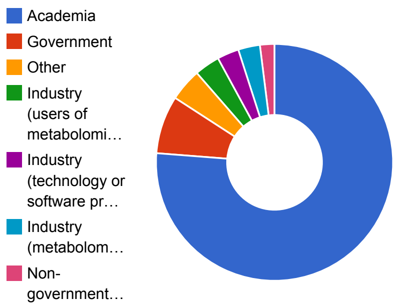

2. What type of work do you do?

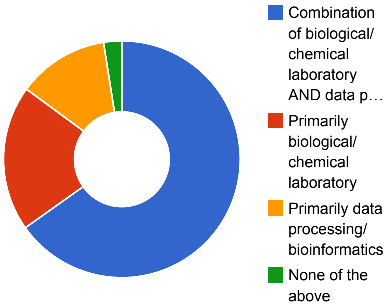

3. Country of Responder

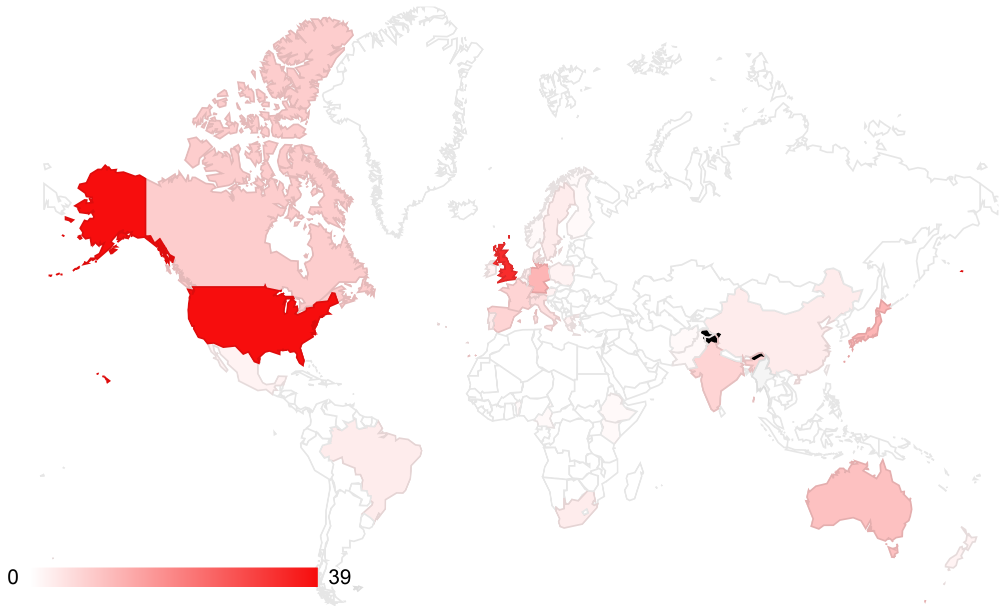

4. Job Position

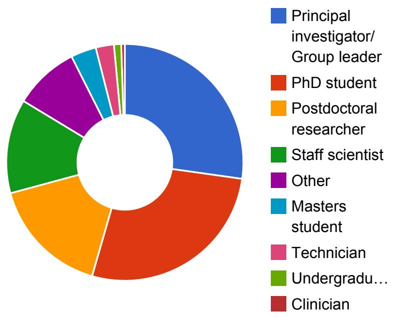

5. Years of Experience

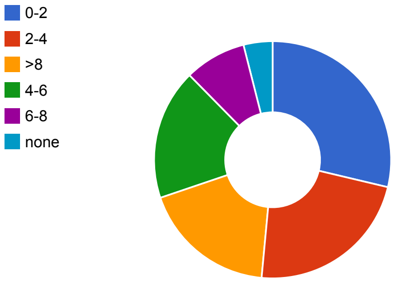

6. In what area(s) of science are you currently applying metabolomics?

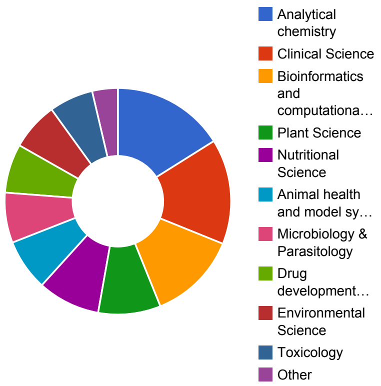

7. What type(s) of samples do you work with?

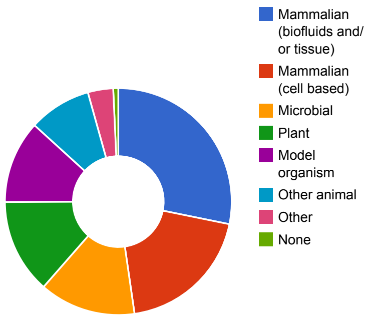

8. What type of metabolomics experiments are you performing?

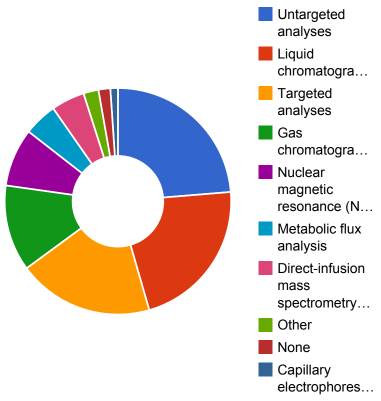

9. What type of bioinformatics are you currently performing?

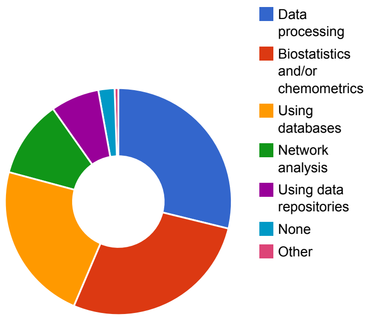

10. What programming skill(s) and language(s) do you use?

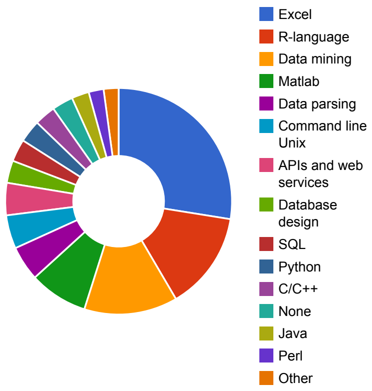

Supplement: Supplementary file 2 — Supplementary material 2 (PDF 146 kb) [file 11306_2015_815_MOESM2_ESM.pdf]
