# Supplementary material for: Training needs in metabolomics
Source: Metabolomics. 2015 May 29;11(4):784–6. doi: 10.1007/s11306-015-0815-6 (PMC4475540; doi:10.1007/s11306-015-0815-6)

Metabolomics Survey - Training Needs (n=202)

1. In what *biological/chemical laboratory* related topic(s) would you like to receive training?

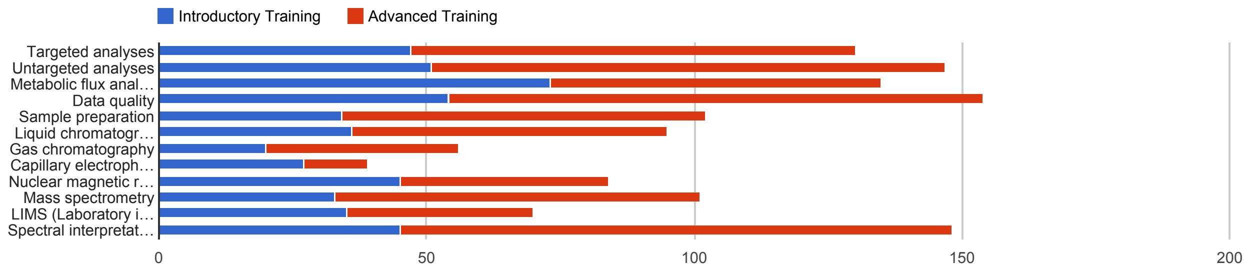

2. In what *bioinformatics* related topic(s) would you be interested in receiving training?

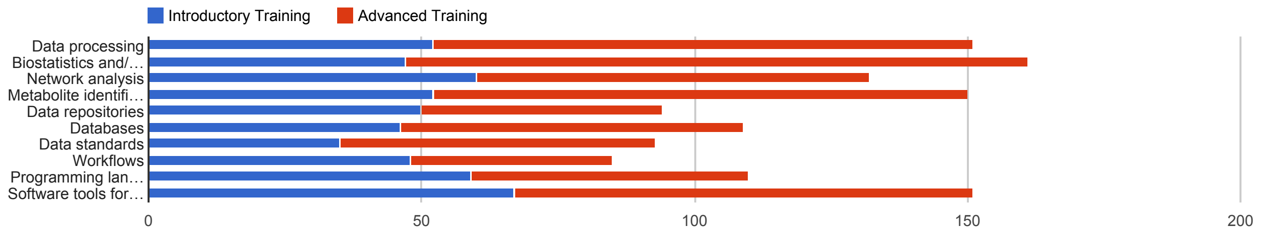

3. In what *programming* related topic(s) would you like to receive training?

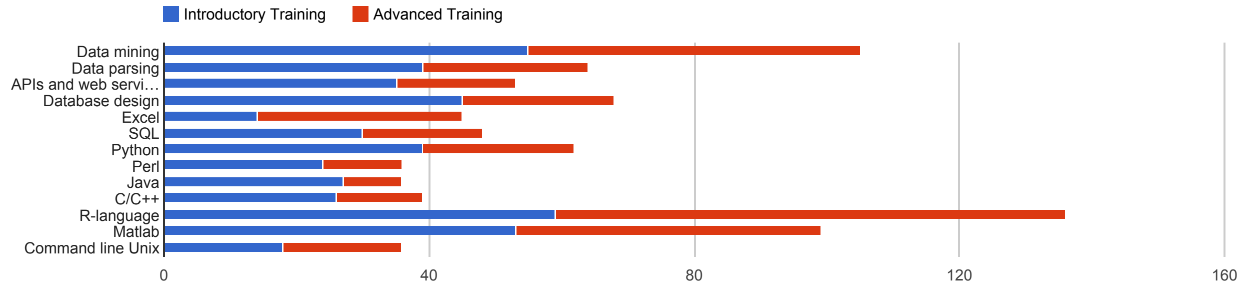

4. What databases do you routinely use?

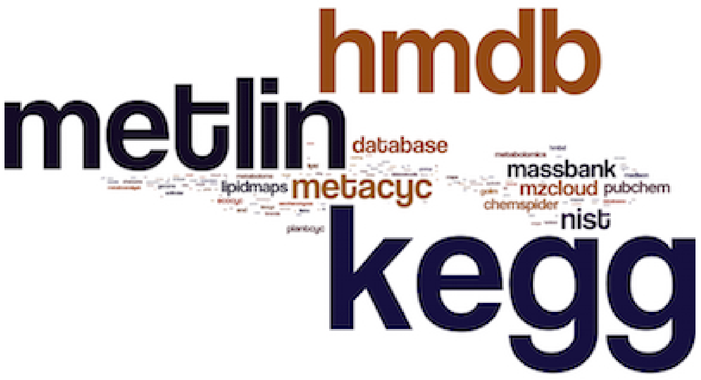

5. What data repositories/databases/tools would you like training on?

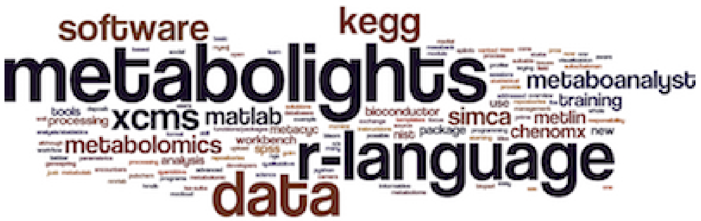

Supplement: Supplementary file 3 — Supplementary material 3 (PDF 200 kb) [file 11306_2015_815_MOESM3_ESM.pdf]
